# Indigestible Substances

**Published:** 1875-02

**Authors:** 


INDIGESTIBLE SUBSTANCES.—It is wonderful to notice the impunity with which all sorts of foreign bodies can be swallowed. Nature seems to take care of almost anything which can be disposed of. A man swallowed a fork last summer, a good-sized dinner-fork, and it has never been heard from since. Insane and idiotic persons have swallowed almost everything except cooking-stoves.

Among the very indigestible and uncomfortable items swallowed, we know of fifteen gold medals, hairpins innumerable, 175 coins, a shoe buckle, nine inches of a sword blade, very sharp scissors, eighty keys, a baby's bottle, a castor, an entire set of dominoes, 100 pennies, a flute four inches long, a glass phial, thirty-five knives, a clay pipe, from 1,400 to 1,500 pins, a bar of lead weighing a pound, and a whetstone. One stomach which was cut open after death contained fifty-two different objects, weighing altogether one pound ten ounces. Among them was a part of the hoop of a barrel, nineteen inches long and one inch wide. It is a remarkable fact that the cases of death caused by the presence of foreign bodies in the digestive tubes are less numerous than might be expected. Out of 163 cases, only ten deaths occurred. To these must be added two deaths after operation, making altogether twelve, or seven three-tenths per cent. There appears, therefore, to be no great cause for the friends to be over anxious in these cases, but to remember that unless there should be some complications in the general health, or some special indication, it will be as well not to interfere, and above all things not to permit the operation known as gastrotomy. This operation has often been successfully performed, once, lately, in order to extract a bar of lead 10 inches long, and weighing 1 pound; and again to do the same with a bar of lead nearly 12 inches long, and weighing more than 9 ounces. In both those cases the symptoms were very serious, comprising violent pains in the stomach, twitchings along the vetebral column, sickness, and general prostration. The foreign bodies could not be felt through the abdominal walls, but the surgeons decided upon performing the operation, thinking that the sufferers had no chance of relief by expulsion. The success of the operations was fortunately complete.

---

QUININE.—Doctors who are in the habit of flying to opium for the purpose of relieving pain, will be glad to know—what the schools may have failed to teach them—that sulphate of quinine, so much relied on as a tonic, will afford relief in most cases of acute rheumatism and neuralgia. The nature and effect of this vegetable salt are becoming better known year by year. Its influence upon the heart's action is very marked. It invariably diminishes the pulsations of the heart even in the healthy subject. Its power over malarial fevers is thought to be owing to its anti-putrid effect, as exhibited in its poisonous action upon animalcules. The processes of putrefaction and fermentation are quickly arrested by quinine, and many disease germs are rendered incapable of development under its action. It reduces the heat of the body, and therefore lessens oxidation in fevers. If taken prior to severe exercise, the perspiration is lessened, and the temperature lowered. This effect lasts about six hours, though all results of copious doses do not disappear for a couple of days.

---

The moral condition of the world depends upon three things—truth, justice and peace.

